# Does a preoperative cone beam CT reduce complication rates in the surgical removal of complex lower third molars? A retrospective study including 486 cases

**DOI:** 10.1186/s13005-021-00271-5

**Published:** 2021-08-14

**Authors:** Jan C. Klatt, Tony Sorowka, Lan Kluwe, Ralf Smeets, Martin Gosau, Henning Hanken

**Affiliations:** Department of Oral and Maxillofacial Surgery, University Medical Center Hamburg-Eppendorf, Martinistr. 52, 20246 Hamburg, Germany

**Keywords:** Cone-beam computed tomography, Radiography, Third molar, Extraction, Inferior alveolar nerve

## Abstract

**Backround:**

This study was designed to analyse the value of preoperative Cone Beam CTs (CBCT) prior to the surgical removal of complex lower third molars. Furthermore, the aim was to assess injuries to the inferior alveolar nerve (IAN) bundle and postoperative neurological disorders depending on the position of the lower third molar and the inferior alveolar nerve bundle.

**Methods:**

In this retrospective examination preoperative Cone Beam CTs and Orthopantomographs (OPT) of 324 patients were analysed concerning the location of the lower third molars in relation to the mandible and the inferior alveolar nerve bundle. Surgery protocols of all patients who underwent the surgical removal of at least one complex lower third molar were analysed concerning patient data, length of surgery, intraoperative haemorrhage, intraoperative exposure of the inferior alveolar nerve bundle, postoperative swelling and postoperative neurological disorders. The data was then compared to data from international studies.

**Results:**

In all 324 patients a permanent neurological damage was not found. Temporary neurological damage was recorded in 13 cases (2.6%). A caudal nerve position with no measurable distance to the root of the lower third molar was associated with the highest risk of a temporal neurological damage. A vestibular touching nerve route also correlated with postoperative sensitivity impairment. If a mesioangulation (Winter) or a Pell and Gregory Type IIIC appears in the OPT, risk of neurological damage is at its highest.

**Conclusions:**

Three-dimensional radiographic imaging, in our patient group, does not significantly affect the risk for complications during the surgical removal of complex lower third molars. Therefore, it should only be utilized for risk assessment, especially in cases of symptom-free lower third molars.

A preoperative orthopantomogram still can be accepted as standard for radiographic imaging.

An intraoperative exposure of the IAN bundle does not necessarily predict simultaneous neurological damage. Exposure of the IAN bundle is no indication for a discontinuation of the surgery.

## Background

The surgical removal of lower third molars is one of the most common procedures in dentomaxillofacial surgery and has been done for over a century [1]. The mostly feared complication during osteotomy is the damage of the inferior alveolar nerve (IAN) bundle with a permanent neurological damage. So the route of the IAN to the third molar root is very important for the operation plan. When the IAN have tight contact to the roots of the third molar the Coronectomy can be a possible choice for the operation to provide neurological deficits. It is also possible to cut the third molar in different pieces to protect the IAN bundle. The more precise the preoperative informations about the course of the IAN is the better the operation can be planed.

While almost all parameters, such as indication, operation technique and perioperative medication have changed and developed over time, dental radiology has improved significantly within the last ten to 15 years [2]. At the present day, applications for CBCTs are increasing while size and prices of CBCT-devices are decreasing. In maxillofacial surgery, the use of CBCTs has helped reduce intraoperative and postoperative complications significantly [3, 4]. The possibility of three-dimensional reconstructions of a patient’s mandible can be tempting and lead to the conclusion of skipping a two-dimensional imaging technique, such as the Orthopantomograph (OPT) right from the start [5].

Many authors and dental societies have developed guidelines and treatment plans for the surgical removal of mandibular third molars [6]. Guidelines for the clinical use of CBCT diagnostics have been published as well, however, some of these guidelines appear to be contradictory in terms of indication and contraindication [7, 8].

The benefit of a preoperative CBCT imaging might be the possibility of a more determined treatment plan and a more precise risk assessment for complex cases. Studies have proven that the CBCT changes the surgeon’s surgical approach [9]. A more differentiated preoperative diagnostic analysis and adapted surgical approaches should consequentially lead to a reduction of complications, and therefore to a reduced number of damaged IAN bundles. Also the stage of development of the third molar roots can have a positive effect on reducing the number of damages to the IAN bundle. Therefore a Germectomy can reduce the risk of neurological damages to the IAN bundle [10].

The aim of this study was to analyse the value of preoperative Cone Beam CTs (CBCT) prior to the surgical removal of complex lower third molars. In particular, the goal was to specify its value in terms of reducing the risk for injury of the inferior alveolar nerve (IAN) and postoperative sequelae like neurological deficiencies. Other studies before demonstrate that CBCT imaging brings no advantage in reducing the risk for a injury of the IAN compared to the less detailed OPT examination [11–14].

We hypothesize that the use of a preoperative CBCT results in a lower amount of iatrogenic injuries of the alveolar inferior nerve bundle and therefore in a lower amount of postoperative neurological disorders for complex third molar removals.

## Materials and methods

In this retrospective evaluation our group of patients were gathered from a dental private practice in Leipzig, Germany. To be included into this group, patients were to be older than 14 years of age and of good general and mental health. In addition to a preoperative OPT, a preoperative CBCT had to be present. Indication for CBCT imaging was drawn from the OPT. The OPT had to show radiologic signs, indicating an increased risk of damage to the inferior alveolar nerve. These signs are the darkening of the third molar root, abrupt narrowing of the root, interruption of the white line of the IAN canal, displacement of the IAN canal by the root and abrupt narrowing of at least one of the white lines representing the IAN canal in proximity of the third molar root [15–17]. The evaluation of the preoperative OPT and CBCT where accomplished by six different Oral surgeons with different skill levels, who standardized the X-Ray examination before the evaluation. During the evaluation a permanent calibration process take place to reduce the bias.

At least one mandibular third molar had to be removed during surgery. Surgery was performed using a standardised removal technique (buccal approach). All teeth were removed by osteotomy with removal of bone and sectioning of the teeth because of the difficult position of all teeth near to the IAN.

Exclusion criteria were a strongly reduced general or mental health, a pre-existing neurological disorder and the simultaneous extraction of teeth other than the third molars during surgery. OPT diagnostics were done using an Orthophos XG® (Dentsply Sirona, Wals bei Salzburg, Austria, voltage 69 kV, current 15.0 mA, exposure time 14 s). The CBCTs were done using an Accuitomo® F 80 (J. Morita Mfg. Corp., Kyoto, Japan, voltage 90 kV, current 5.0 mA, exposure time 9–18 s, FOV 55–58%). All following measurments of CBCTs then were accomplished with the I-Dixel Software from Morita (J. Morita Mfg. Corp., Kyoto, Japan).

**Fig. 1:**
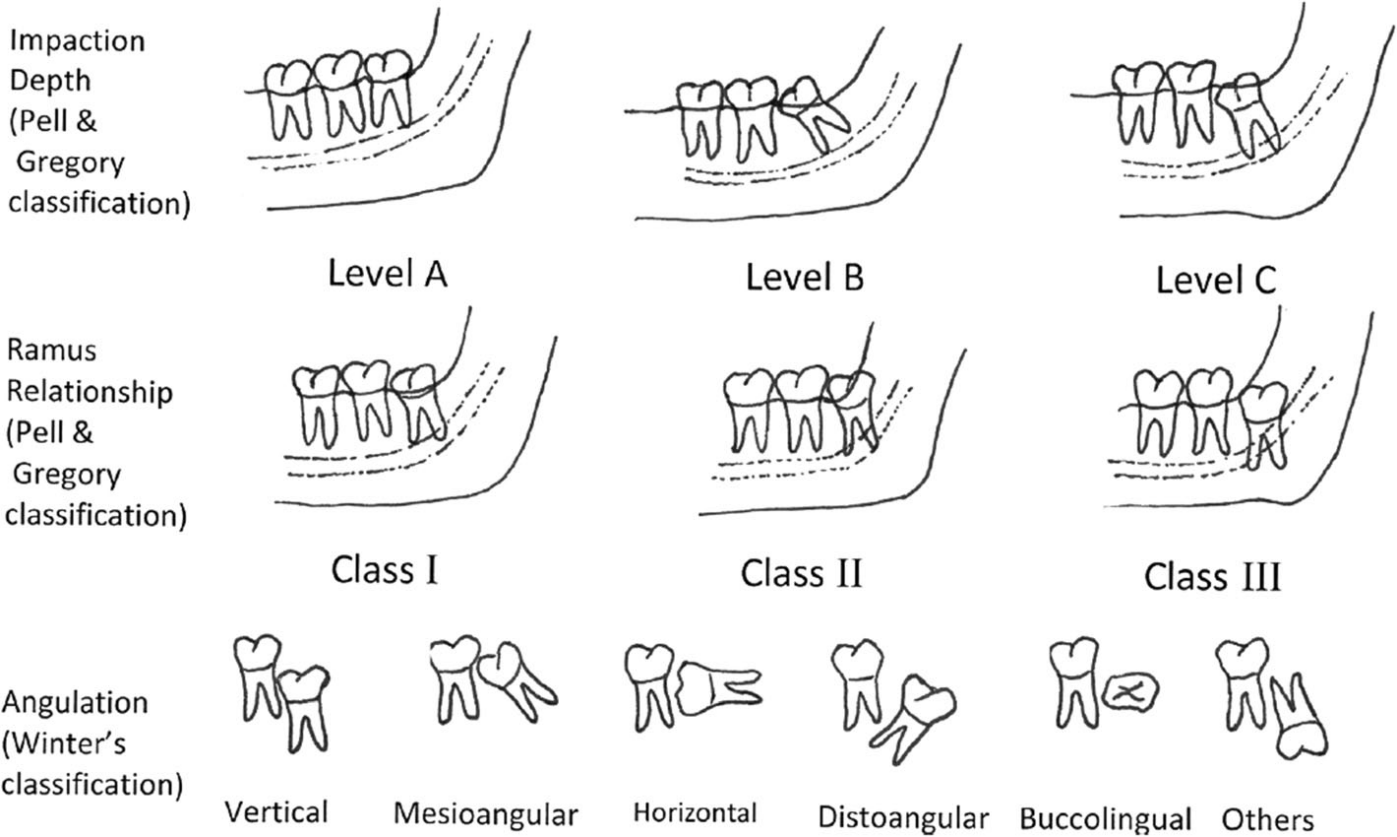
shows the classification of Pell and Gregory and the classification of Winter [15]

The preoperative OPTs were analysed using the internationally widely spread classification of Pell and Gregory as well as the classification of Winter (Fig. 1) [18–20]. Evaluating the CBCT, it is common to describe the position of the tooth in terms of impaction (impacted or not impacted) and angulation (mesial, vertical, distal and horizontal). The relationship between the IAN and the lower third molar can be described by characterising the nerve canal in relation to the tooth in terms of vestibular, caudal and lingual. Since the objective is to quantify the risk of surgery in relation to the nerve’s position, it appears reasonable to subdivide the position of the nerve into a vestibular, vestibular touching, caudal, caudal touching, interradicular, lingual and a lingual touching nerve route. ‘Touching’ includes all cases with no measurable distance between the nerve canal and the lower third molar. The CBCT was used to measure the distance of the IAN canal to the apex of the lower third molar. To receive detailed data, we analysed patient age, gender, intraoperative factors, such as procedure length, exposure of the IAN and the postoperative outcome concerning the need of medication, swelling and impairment of nerve sensibility. Procedure length was divided into the following three groups: short - less than 10 minutes, moderate - between 10 and 25 min, and long - more than 25 min. Postoperative healing was also divided into three groups, which are: normal - no swelling/pain in less than 7 days, delayed - no swelling/pain in less than seven to 21 days, and heavily delayed - no swelling/pain within more than 21 days. It is common to distinguish between a temporary and a permanent neurological damage to the IAN nerve bundle, while in this case ‘permanent’ can be described as altered sensation for more than 6 months. We subdivided the temporary damage into these three groups: short - seven to 14 days, moderate - 14 to 30 days and long - more than 30 days.

## Results

Three hundred and twenty-four patient files matched the inclusion criteria. These patients underwent surgery between February 14th, 2006 and December 18th, 2014. During this time six oral surgeons performed the procedure and removed a total of 486 lower third molars. Three of these surgeons had more than 25 years of surgical experience. The other three surgeons had less than 5 years of surgical experience. The resolution and quality of CBCTs and OPTs in our study were sufficient for the analysis of the position of the IAN canal to the roots of the third molar. In one case the nerve position could not be described, because a pathological follicular cyst made classification unreliable. There has been no atypical fracture in our patient group. The points postoperative infection and bleeding have not been measured.

### General results / epidemiology

**Fig. 2:**
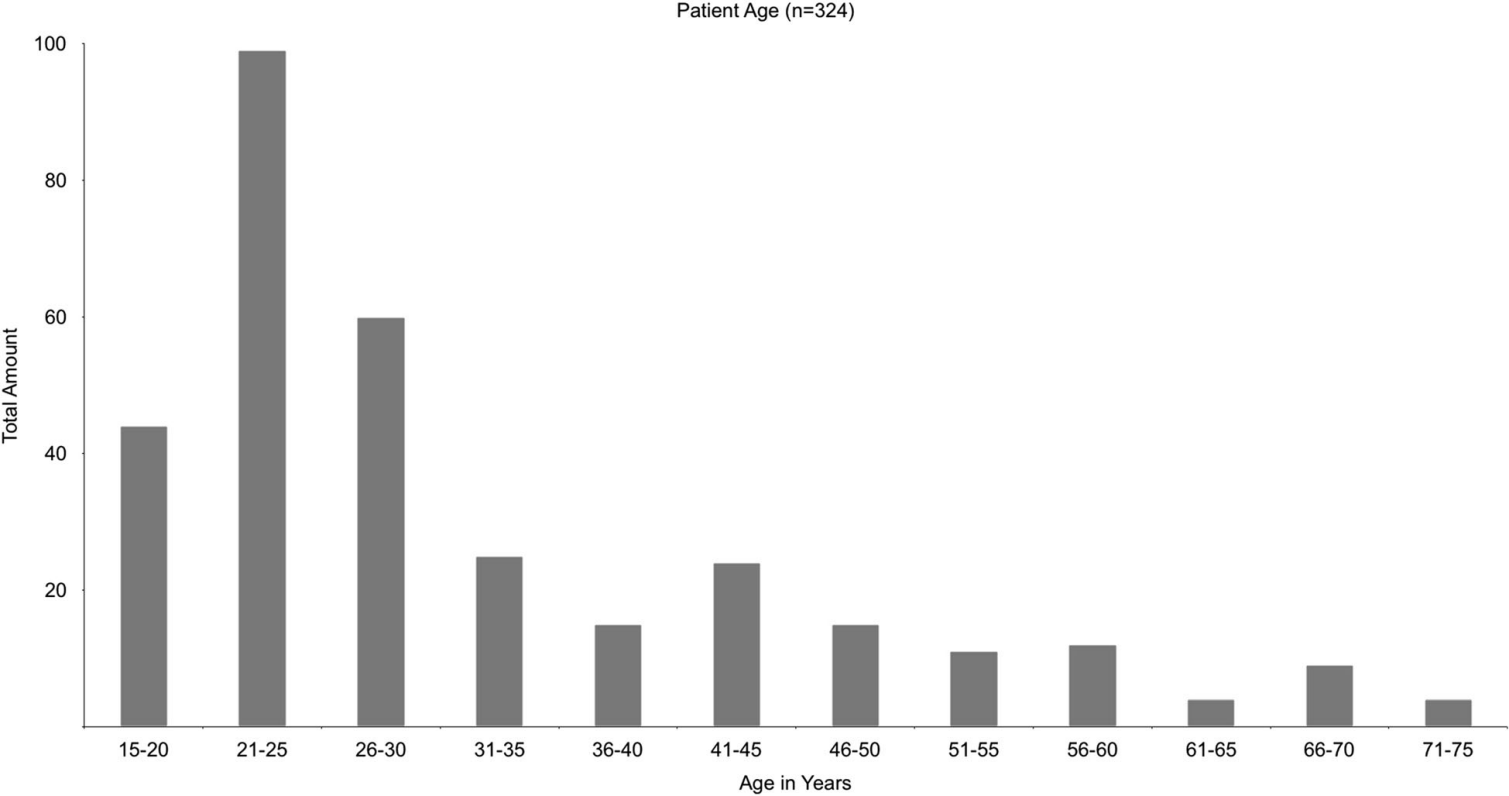
shows the age distribution for all 324 patients

The patient group included 324 patients of which 185 are females and 139 males. At date of surgery, patients were between 16 and 75 years old. The mean age was 32.1 years (Fig. 2).

### Intraoperative data

#### Procedure length

Procedure length was measured from incision to suture. 74,5% of all procedures were of moderate length.

#### Exposure of the IAN

The intraoperative exposure of the IAN was documented in 12 cases (2.5%).

### Postoperative data

#### Postoperative swelling

Most patients showed a reduction of pain and postoperative swelling within 7 days (88.6%). Only 27 cases showed a delayed healing process, showing postoperative pain for more than 21 days (8.1%).

#### IAN disorders

**Fig. 3:**
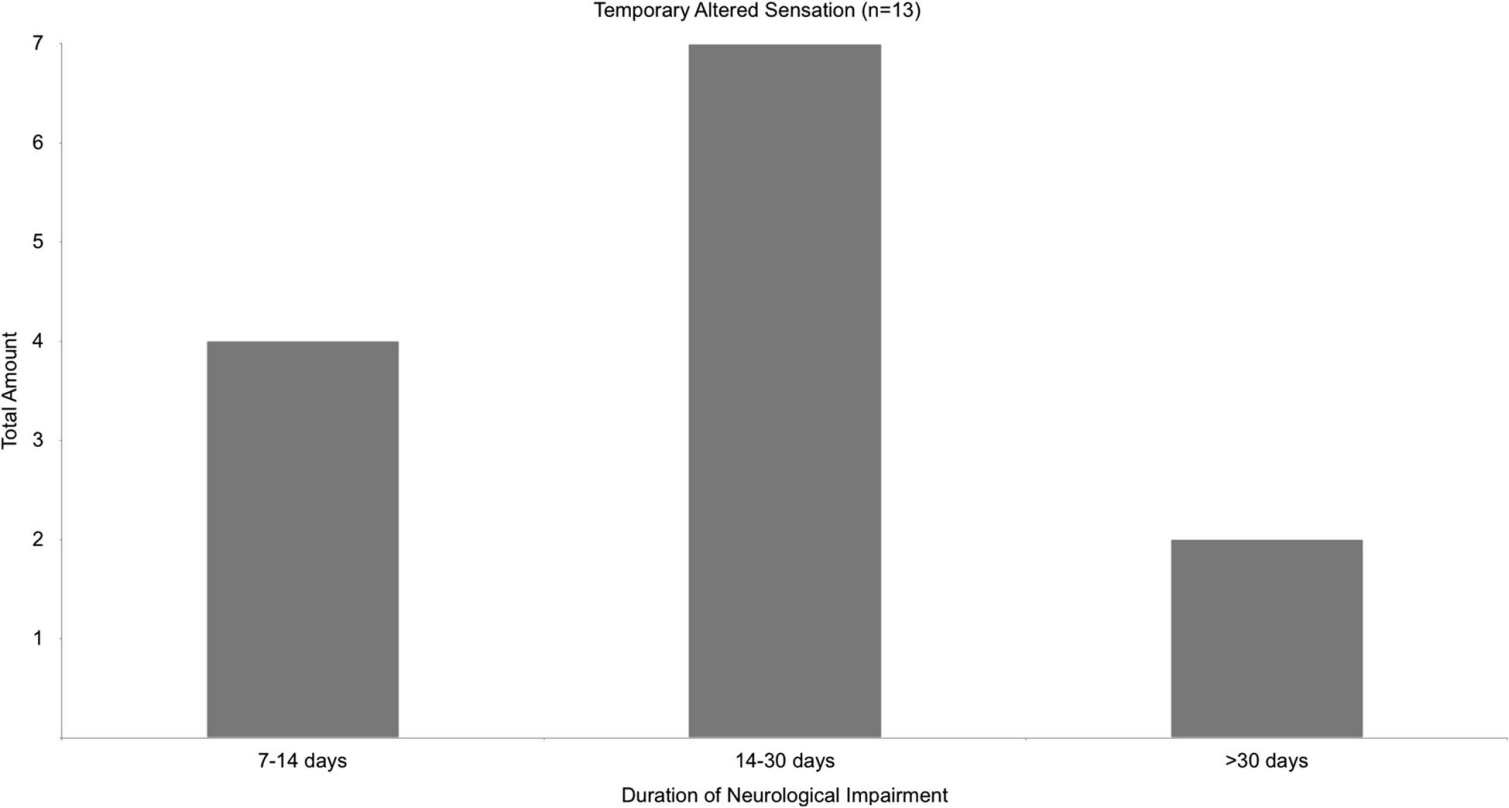
shows the amount of cases with temporary altered sensation depending on the duration of neurological impairment

Permanent neurological damage, defined as altered or missing sensation within the innervation area of the IAN for more than 6 months, was not reported. Postoperative anaesthesia was always of temporary nature, turning into hypoesthesia within days. Temporary altered sensation was reported in 13 cases (4.0%). The majority of patients with altered sensation regained full neurological function within 30 days or less (84.6%, Fig. 3).

Intraoperative exposure of the IAN correlated with a temporary reduced sensory function in two cases (15%).

### Radiology data

#### OPT diagnostics

##### Pell and Gregory

**Fig. 4:**
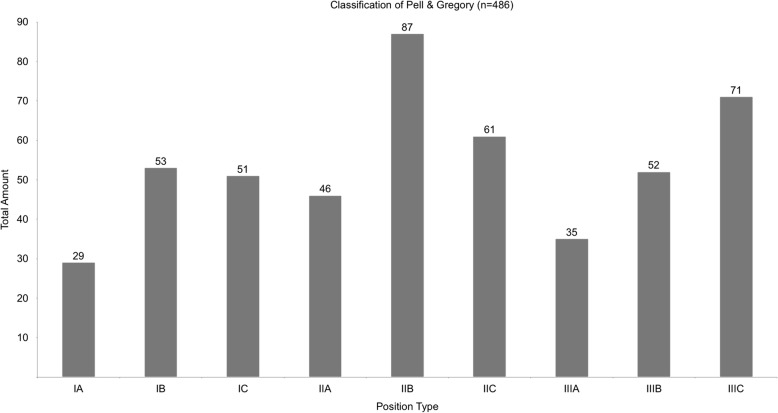
shows the position types for all 486 teeth in the classification of Pell and Gregory

**Fig. 5:**
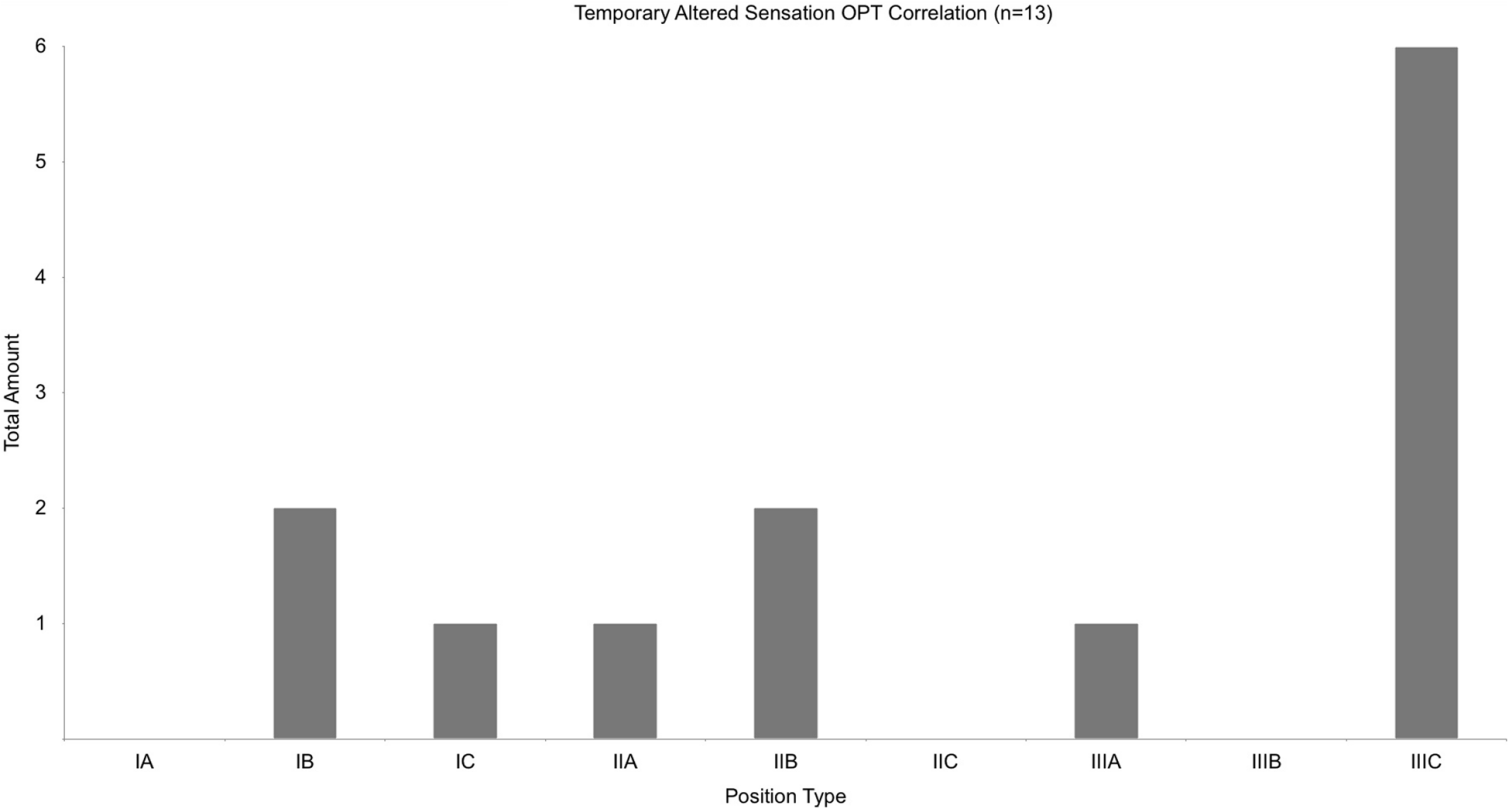
shows the distribution of position types after Pell and Gregory for all cases with temporary altered sensation

The tooth positions, defined by Pell and Gregory, distribute among the patient group as shown in Fig. 4. Figure 5 shows the classification of Pell and Gregory for all cases with postoperative sensory disorder. A significant number of these lower third molars were categorised Type IIIC (42%).

##### Winter

**Fig. 6:**
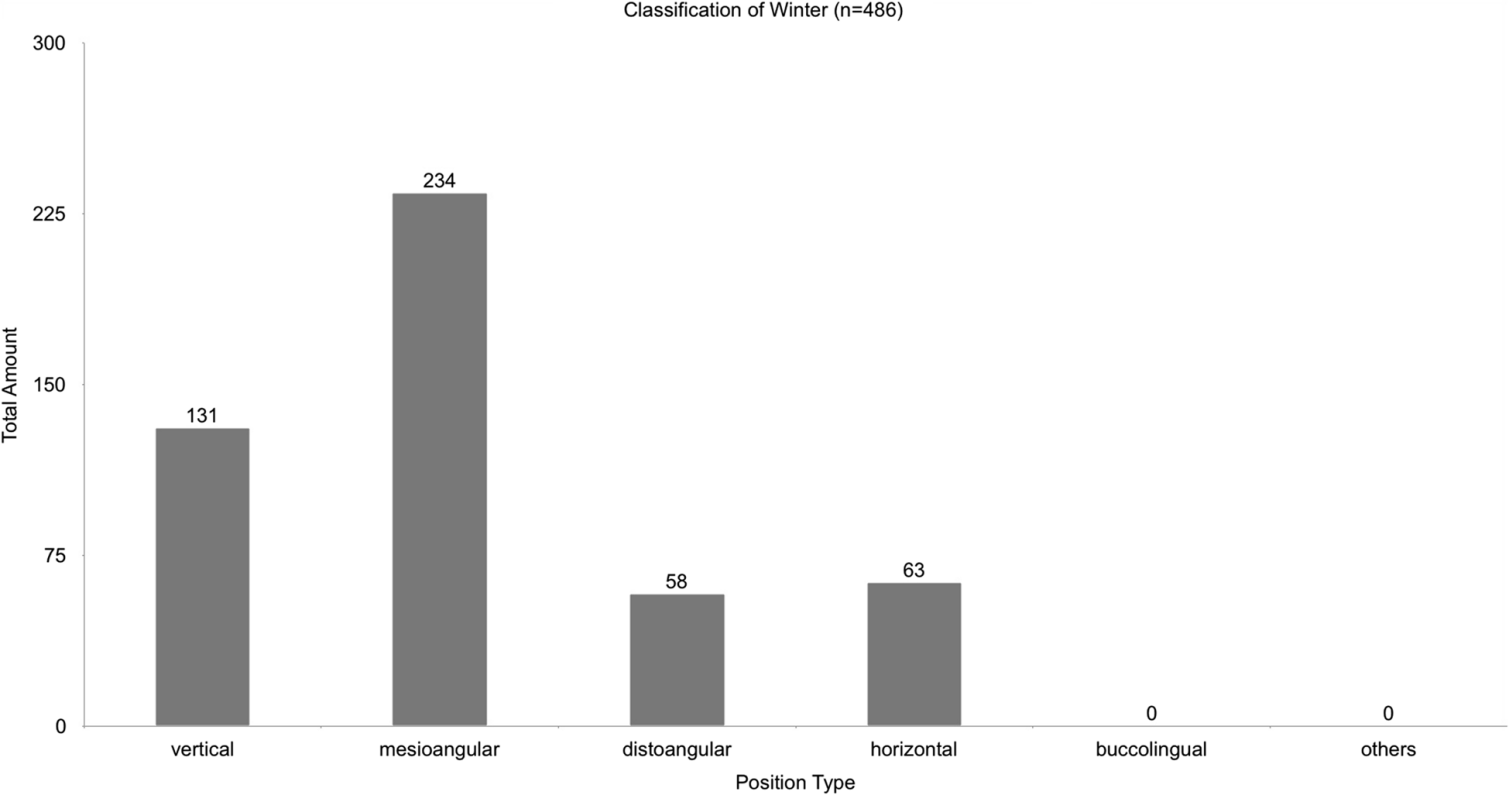
shows the position types for all 486 teeth in the classification of Winter

**Fig. 7:**
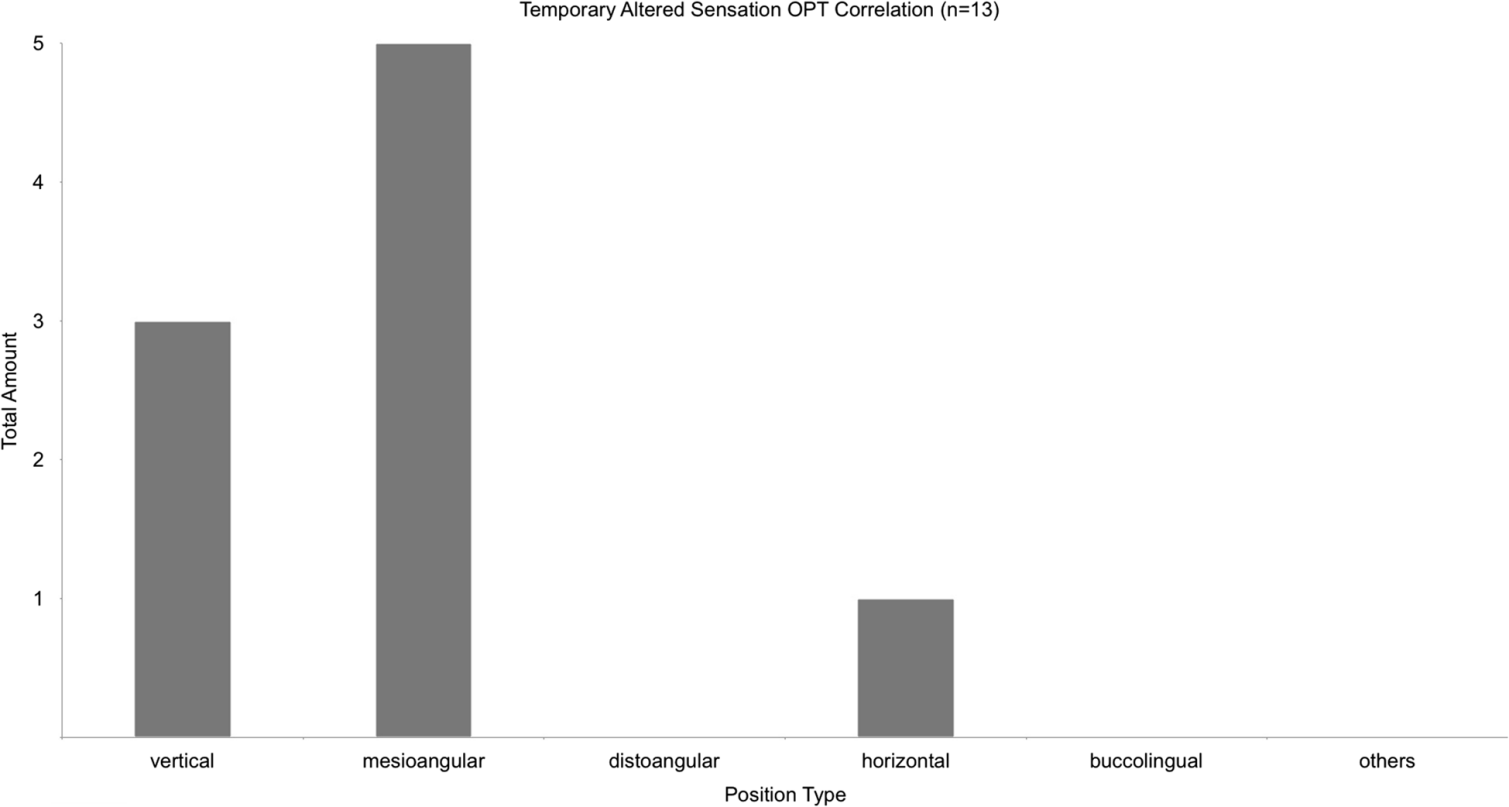
shows the distribution of position types after Winter for all cases with temporary altered sensation

The majority of lower third molars removed were mesio-angulated (48.1%). Figure 6 shows the classification of Winter for all cases. Figure 7 shows the classification of Winter for all cases with postoperative sensory disorder.

#### CBCT

##### Nerve position

**Fig. 8:**
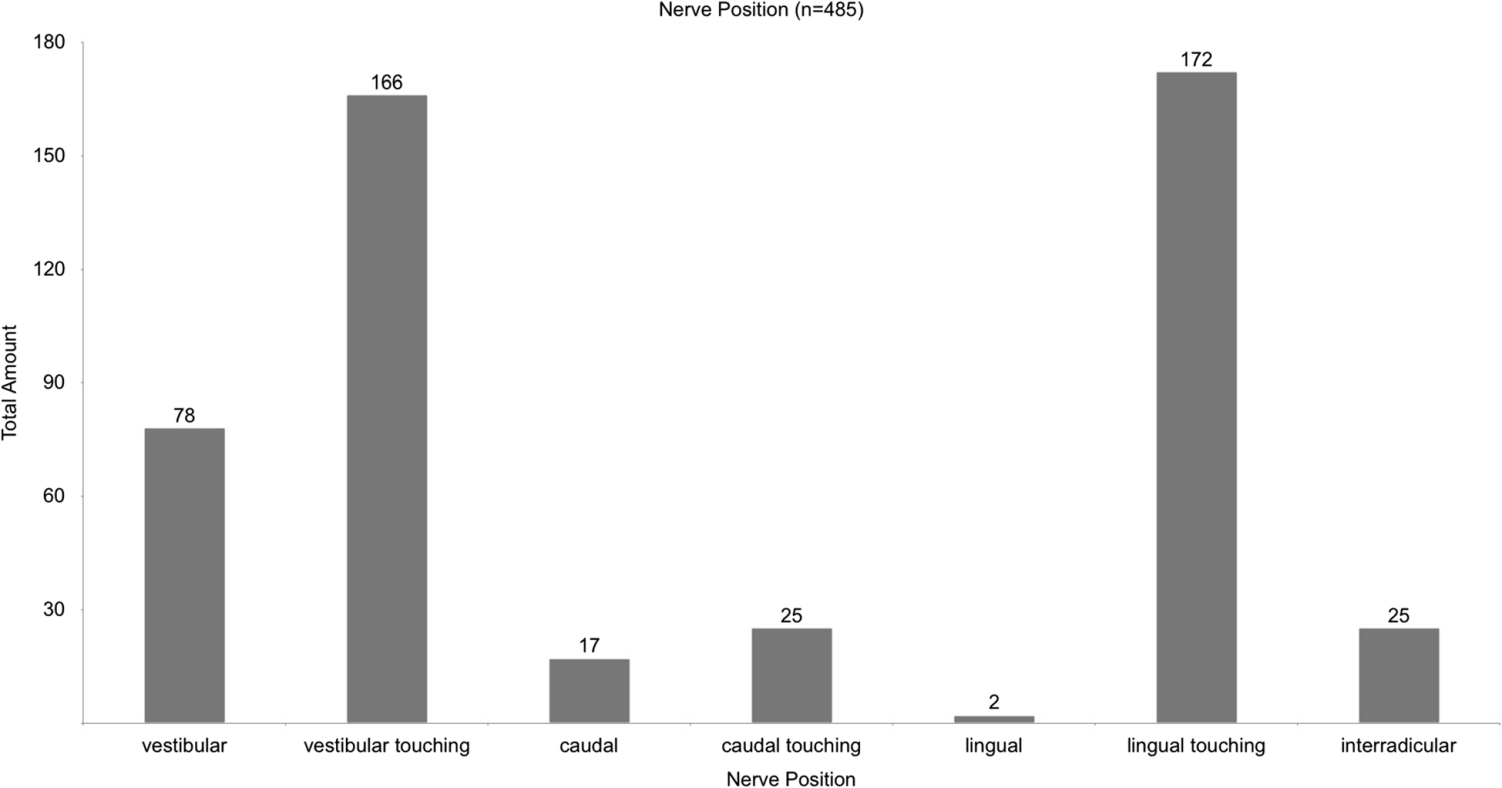
shows the distribution of nerve positions extracted from the CBCTs

The CBCT analysis showed, that most nerve positions were vestibular touching (166 cases, 34.2%) or lingual touching (172 cases, 35.4%). A significant amount of nerve positions was touching the lower third molar (388 cases, 80%). A lingual nerve position was in direct contact to the lower third molar in 98.8% percent of the time. An interradicular nerve position was found in 25 cases (5%). Figure 8 shows the distribution of nerve positions.

##### IAN distance to mandibular third molar

The average distance of the IAN canal to the mandibular third molar was 0.3 mm. The maximum distance was 5 mm.

##### IAN distance to horizontal impaction (winter)

In 62 cases (12.7%) the lower third molar was horizontally impacted. Of these 62 teeth, 57 (91.9%) had a nerve position touching the apex of the lower third molar. Nerve injury occurred in only one of these 62 cases, resulting in temporary altered sensation.

##### IAN distance to clinical IAN exposure

In all reported cases with clinical IAN exposure preoperative CBCTs revealed a nerve position touching the dental roots. Five cases with clinical IAN exposure showed a caudal touching nerve position (41.7%). An interradicular course of the IAN was reported in two cases with intraoperative IAN visibility (16.7%).

##### CBCT diagnostic to neurological disorder

**Fig. 9:**
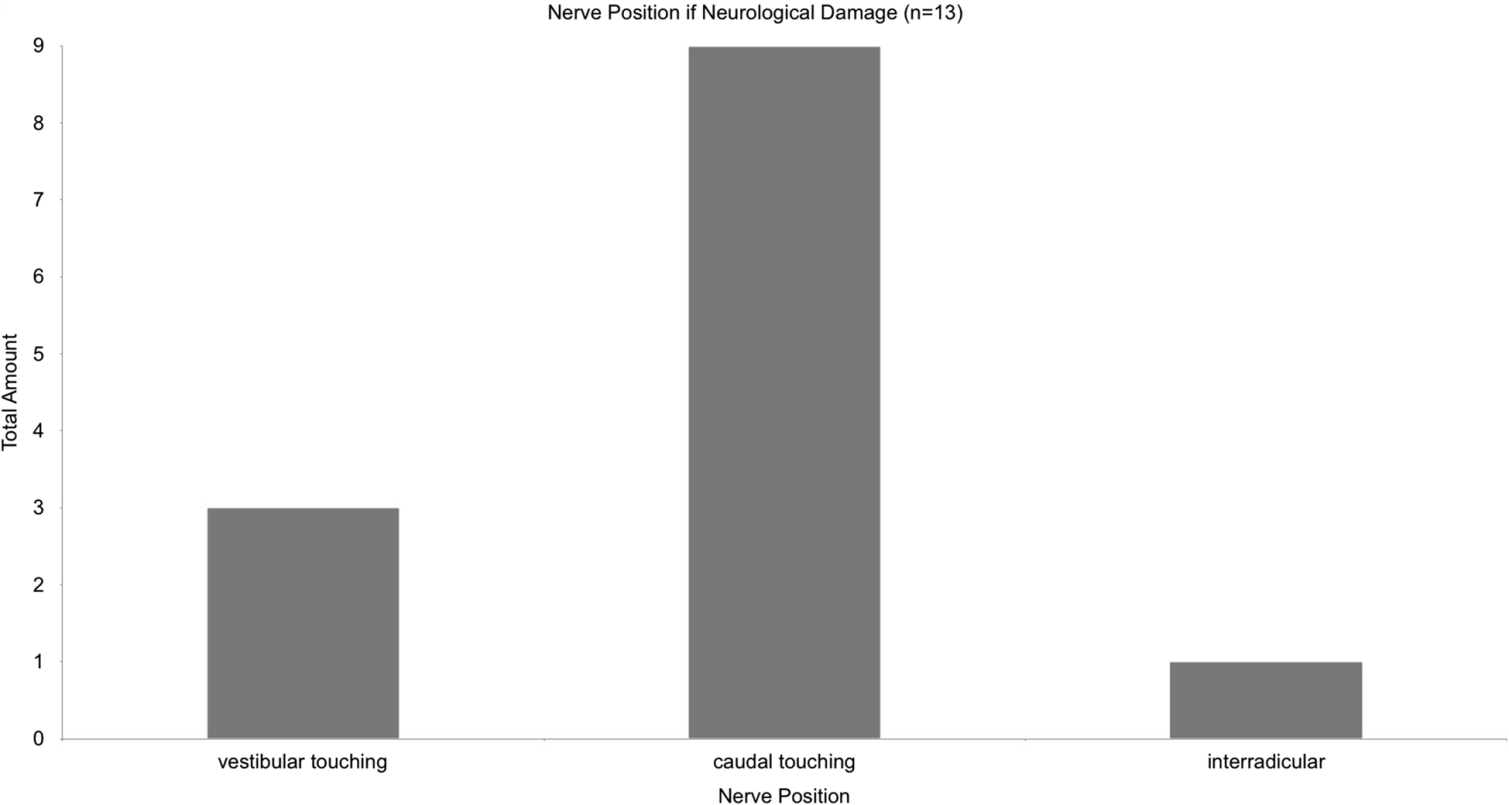
shows the distribution of nerve positions for all cases with temporary altered sensation

Within the group of 13 patients with postoperative altered sensation, a significant amount of nerve positions was caudal touching (nine cases, 69%, Fig. 9). Three cases were vestibular touching (23%) and only one was interradicular (7%). In all cases the CBCT measured distance between the IAN and the lower third molar was 0 mm.

## Discussion

The purpose of this study was to find a correlation between the use of a CBCT prior to the surgical removal of lower mandibular third molars and the postoperative outcome in terms of neurological disorders. The analysis of this correlation is necessary, because surgeons need reliable data when informing the patient about possible risks of a surgical procedure. We hypothesised that utilizing preoperative three-dimensional radiographs would lower the amount of iatrogenic nerve injuries. In our study, 13 out of 324 patients (2.6%) suffered from neurological damage after surgery. To put this number into perspective, we searched international literature for studies, stating the amount of iatrogenic nerve injuries during the removal of lower mandibular third molars. Regardless of the complexity of the extraction, literature shows a wide range in surgery risk varying between 0.8 and 8.4% [2, 21]. In 2006, Jeries analysed the occurrence of neurological impairment during lower mandibular third molar surgical in a prospective study with a comparably high number of patients (1087 cases). Patients in this study only underwent two-dimensional imaging in form of an OPT. The occurrence of temporary neurological disorders was stated at 4.1%, while permanent damage occurred in 0.7% [22]. Multiple study parameters of Jeries’ were equal or similar to our study, but it has to be considered that the average difficulty of the lower third molar extraction in Jeries’ case is lower than in our study, since in our case all two-dimensional radiographs showed signs of increased risk to the IAN. In our opinion, the amount of nerve injuries in complex cases with a preoperative CBCT (2.6%) is comparably high, when looking at a risk of 4.8% for cases with only an OPT.

The clinical exposure of the IAN bundle during the surgical removal of lower third molars depends on many factors, e.g. maximum mouth opening, intraoperative bleeding and nonetheless the location and route of the IAN. In the past, authors have correlated intraoperative exposure of the IAN and neurological damage [5]. Our study presents, that there is no significant correlation between both. Only in two cases with postoperative neurologic deficiencies the IAN was visible to the surgeon. One of these nerve routes was caudal, the other one was interradicular, which can be described as a subtype of caudal. It appears logical, that a surgeon rather takes notice of an IAN if its route is caudal (touching) and that a vestibular nerve can rarely be seen due to the perspective of the surgeon. Leung states, that a neurological deficit is 14.9 times more likely when the IAN bundle is exposed during the procedure [16]. The review includes the buccal approach, lingual split technique and coronectomy. The surgical approach also has great impact on a possible exposure of the IAN bundle. It is necessary to specify the surgical approach when searching for a possible correlation with complications.

A major factor influencing the outcome of a study is the surgeon. His experience and skill level as well as his physical and mental state influence every possible statistic. In this study, surgery was performed by six different oral surgeons with varying skill levels and experience.

Postoperative swelling as well as the reception of pain are extremely difficult to objectify. In this study it was necessary to rely on patient documentation only. To achieve a more distinct and objective picture of postoperative swelling and pain, a prospective study with predesigned standardised questionnaires and medical reports is necessary.

Damage to the IAN can result in anaesthesia, paraesthesia, pain, or a combination of the three [23, 24]. Some patients do not recognise the hypoesthesia until the sensory field is objectively tested. To objectify postoperative hypoesthesia, the receptive field of the IAN was tested on every patient 1 day after surgery by testing the sharp-dull discrimination and two-point discrimination of the receptive field. The analysis of pain reception, postoperative swelling and tests on the receptive field add up to a detailed data pool that allows a comprehensive interpretation.

The lack of accuracy when using only an OPT for preoperative diagnostics has been described by many authors in the past [5, 25–27]. Before CBCT diagnostics were available, radiological signs in two-dimensional diagnostics were indication for a CT-Scan. Even though CT diagnostics have been available since 1972, clinicians in dentistry have not used computed tomography routinely, mainly, due to an initial lack of access to the machines and due to the comparably high radiation exposure [28]. The production of smaller and more affordable CBCT devices in the early 2000s gave dentists and maxillofacial surgeons the ability of cost effective three-dimensional diagnostics combined with a low radiation exposure for patients and medical staff [29–32].

Today, multi-detector computed tomography (MDCT) and low-dose protocols result in much lower radiation exposure during CT diagnostics. The effective dose of a MDCT can be as low as 0.15 mSv, dropping below effective doses of some CBCT devices. Still, with a sub-millimetre spatial resolution, the CBCT is the preferable imaging technique for dentoalveolar diagnostics [26].

In our study preoperative CBCT does not reduce the risk of postoperative neurological damage. But the more detailed information gathered by CBCTs enables the surgeon to choose the appropriate surgical approach, what leads to an adjusted and therefore less invasive operation with less operation time.

Compared to about 22 μSv effective dose of an OPT, CBCTs still cause much higher radiation exposure than two dimensional radiographs, which is why CBCT should not be considered for standard diagnostics, but only if the OPT shows risk factors for IAN damage [25, 33, 34].

If theire is a postoperative loss of sensory function of the IAN Bundel the magnetic resonance imaging (MRI) ist still the best examination for proving a IAN bundle damage [35].

The complete removal of a lower third molar by buccal approach is probably the most common, but not the only therapy option, when indication for surgery is confirmed. Several authors proclaim coronectomy as the approach of choice, when the lower third molar is in proximity of the inferior alveolar nerve [36–38]. While the benefit of coronectomy versus complete extraction is controversially discussed in literature, indications and contraindications of the procedure have been defined clearly [2, 36]. One contraindication for a coronectomy is the horizontal rotation of the lower third molar. Our data shows, that a horizontally rotated lower third molar (Winter) in proximity to the inferior alveolar nerve (OPT) results in no significant correlation with postoperative sensitivity impairment. It can be concluded, that if contraindication for coronectomy is present due to a horizontal rotation, complete extraction should be the procedure of choice.

The CBCT is also of significance when evaluating the removal indication of symptom-free lower third molars. The indication for surgery should be revaluated if the CBCT shows a caudal touching or interradicular route.

## Conclusion

In our study, permanent damage to the IAN bundle did not occur. Postoperative altered sensation was always of temporary nature. Preoperative CBCT does not reduce the risk of postoperative neurological damage, yet, it may lead to an adjusted and therefore less invasive surgical approach [32].

Standard diagnostics should always include an OPT. Due to radiation protection, the CBCT should not be used for routine diagnostics.

The Indication for CBCT diagnostics is present, when the surgeon identifies radiological signs that predict IAN damage on the OPT. These signs are the darkening of the third molar root, abrupt narrowing of the root, interruption of the white line of the IAN canal, displacement of the IAN canal by the root and abrupt narrowing of at least one of the white lines representing the IAN canal in proximity of the third molar root [16]. If these signs correlate with a mesioangulation (Winter) or a Pell and Gregory Type IIIC, risk of neurological damage is at its highest.

Evaluating the CBCT, a caudal nerve route with no measurable distance to the root of the lower third molar, predicts the highest risk of neurological damage. A vestibular touching nerve route also indicates a high risk of postoperative sensitivity impairment.

An intraoperative exposure of the IAN bundle does not necessarily predict simultaneous neurological damage. Exposure of the IAN bundle is no indication for a discontinuation of the surgery.

## Data Availability

Datasets obtained or analysed during the current study are available from the corresponding author on reasonable request.

## Abbreviations

CBCT: Cone Beam Computer Tomography
IAN: Inferior alveolar nerve
OPT: Orthopantomograph
MRI: Magnetic resonance imaging
